# Neuroart: picturing the neuroscience of intentional actions in art and science

**DOI:** 10.3389/fnhum.2015.00410

**Published:** 2015-07-23

**Authors:** Todd Siler

**Affiliations:** ArtScience® PublicationsDenver, CO, USA

**Keywords:** intentions, creativity, divergent and convergent thinking, innovating, neuroscience of creativity, neuroscience of art, neuroaesthetics, embodied aesthetics

## Abstract

Intentional actions cover a broad spectrum of human behaviors involving consciousness, creativity, innovative thinking, problem-solving, critical thinking, and other related cognitive processes self-evident in the arts and sciences. The author discusses the brain activity associated with action intentions, connecting this activity with the creative process. Focusing on one seminal artwork created and exhibited over a period of three decades, *Thought Assemblies* (1979–82, 2014), he describes how this symbolic art interprets the neuropsychological processes of intuition and analytical reasoning. It explores numerous basic questions concerning observed interactions between artistic and scientific inquiries, conceptions, perceptions, and representations connecting mind and nature. Pointing to some key neural mechanisms responsible for forming and implementing intentions, he considers why and how we create, discover, invent, and innovate. He suggests ways of metaphorical thinking and symbolic modeling that can help integrate the neuroscience of intentional actions with the neuroscience of creativity, art and neuroaesthetics.

## Introduction

Nature makes everything look simple. But nothing is as complicated as “simplicity”: from jotting down this observation to drawing things my imagination envisions that exemplify what I mean by simplicity. These intentional actions join countless others born from brain dynamics that neuroscientists grope to understand with great ingenuity in laboratory settings (Chatterjee, 2004; Dietrich and Kanso, 2010; De Dreu et al., 2014). Making that essential leap to real-world settings, in order to model the neurobiology of intentionality and creativity in more naturalistic or realistic ways, may be the ultimate methodological and technological challenge of human neuroscience (Jung et al., 2013).

Applying imagination to knowledge gained from empirical studies creates opportunities to develop new methods and tools for discovering the connections between nervous systems, mental processes and patterns of behavior. As the pioneer neuroanatomist Santiago Ramon y Cajal reminds us: “Discoveries are largely a function of the methods used.” Bold goals can catalyze the process.

One paramount goal of the neuroscience of art is to understand the nature of creativity (Runco and Jaeger, 2012; Vartanian et al., 2013). What constitutes a creative process? How it is manifested in the brain? And how it is manifested in art that connects science, technology, engineering, mathematics, and all other forms of disciplinary knowledge? By utilizing a wide array of neuroimaging tools (fMRI, PET, SPECT) coupled with psychometric techniques (Dietrich, 2004; Arden et al., 2010), which help interpret the meanings and implications of brain dynamics gathered from experimental studies, researchers are probing the molecular, cellular, cognitive and behavioral responses to art (Dietrich and Kanso, 2010; Runco et al., 2011; Vartanian, 2012; De Dreu et al., 2014). Moreover, they’re searching the biological and genetic basis of creativity (Reuter et al., 2006; Runco et al., 2011; Jung et al., 2013; Dietrich and Haider, 2014) and novelty seeking (Ebstein et al., 1996; Schweizer, 2006; Mayseless et al., 2013).

In their quest to illuminate common brain dynamics and behaviors underlying artistic creativity and aesthetic experiences (Zeki, 2001; Ramachandran, 2011; Jung et al., 2013; De Dreu et al., 2014; Ticini et al., 2015), neuroscientists are examining the interconnections of art and cognitive science (Epstein, 1999; Freedberg and Gallese, 2007). These studies deepen and bridge our understanding of the *work* of art (Gero and Maher, 1993; Gero, 2002; Cavanagh, 2005) as it relates to the *work* of science (Root-Bernstein and Root-Bernstein, 1999).

## Seeing the Big Picture of Intentional Actions that Connect Art and Science

Understanding intentional acts of artistic and scientific inquiry entails connecting what neuroscience knows about creativity, art, aesthetics, and intentions with an overarching perspective that considers how creative cognition occurs in combination with environmental, social and cultural influences (Amabile, 1982; Csikszentmihalyi, 1988). This perspective advocates practicing integrative thinking, in order to create an integrated neuroscience: one that unifies our collective knowledge of brain-mind processes (Churchland, 1989), utilizing an ArtScience prospective (Root-Bernstein et al., 2011).

There are many compelling, evidence-based theories that describe characteristics of the creative process as they relate to acts of creating, experiencing and appreciating art (Kawabata and Zeki, 2004; Ishizu and Zeki, 2011; Ticini and Omigie, 2013). For example, neuroaesthetics explores various areas of the human cerebrum that are stimulated by these intentional actions. Using functional magnetic resonance imaging (fMRI), the neuroscientist Semir Zeki and his colleagues at the Laboratory of Neurobiology at University College, London are revealing how we all share “common neurobiological processes” which enable us to generate “almost infinite creative variability.” Zeki (2001) theorizes that these processes afford us the ability to “create radically different styles” and forms of artistic expression (Onians, 2008). They also enable us to experience virtually all art forms, including dance and performance art, as “embodied aesthetics” (Cross and Ticini, 2011; Ticini et al., 2015).

Other noted theories analyze features of human creativity that involve decision making (Vartanian, 2011) under the influence of uncertainty and biases (Kahneman et al., 1982). This area of research bares insights into the process of artistic and scientific inquiry and discovery. It prompts me to ponder why and how I value certain aesthetic experiences (e.g., creative boldness, originality and risk-taking) when evaluating works of art and science I admire, such as the Bayesian unified theory of brain dynamics. This behavioral science tool applies a statistical parametric mapping (SPM) instrument for investigating the central nervous system’s ability to manage uncertainty (Doya et al., 2007; Friston et al., 2013). Perhaps, it can provide a more comprehensive view of the unpredictable process of creativity which embodies plenty of uncertainty. The “Bayesian coding hypothesis” suggests that neurons code sensory information *probabilistically* (Knill and Pouget, 2004). Implying, our perceptions, actions, judgements, and decisions can be represented as forms of “probability distributions”. Surely, this tool can also be applied to the causative studies in neuroplasticity, which reveal the neural mechanisms of creative thinking and skills across many domains and intentional actions that change the brain in measurable ways (Fadiga et al., 1995; Pascual-Leone et al., 1995; Doidge, 2007).

## Neuroart Depicting a World of Thoughts, Feelings, Emotions, Experiences, and Ideas

**Figure 1:**
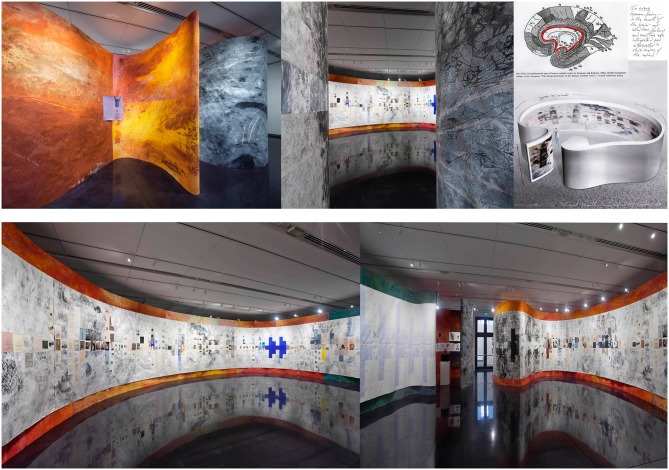
***“The Brain Theater of Mental Imagery* with *Thought Assemblies* emerging from the Limbic System,” (1979–82, 2014).** Mixed media on synthetic canvas with collage elements, 11 ft. × approx. 145 ft. perimeter × 37 ft. diameter. Installation view: CU Art Museum, University of Colorado Boulder (Courtesy of the CU Art Museum. Photo: Jeffrey Wells). (Pictures of “Thought Assemblies” courtesy of The Picower Institute for Learning and Memory, MIT Art Collection).

Experimenting with various methods of discovery defines my work process as a practicing visual artist (~40 years) exploring the nature of creativity. Posing questions about the creative process like a theoretical neuroscientist, I link putative brain processes of creativity with the actions of my unconscious and conscious intentions that I associate with the art-making process (Ramachandran and Hirstein, 1999; Zeki, 1999, 2001). These actions are core to the creative freedom I’ve experienced innumerable times while conceptualizing, designing, creating, and installing my works of art (see Figure 1).

For example, specific neural mechanisms, such the parieto-frontal networks (Jung and Haier, 2007; Gallivan et al., 2011), the fronto-temporal region (Flaherty, 2005), the emotion-related areas involving the amygdala (Vuilleumier and Driver, 2007; Scharpf et al., 2010), hippocampal neurons (O’Reilly et al., 1998; Wilson, 2002), and long-term memory and associative memory networks (Anderson and Bower, 1973; Schacter, 1992, 1996; Cowley and Underwood, 1998), enable me *to know or have a sense of* what I intend to draw (Damasio, 2000)—*before* I’ve physically drawn anything. But I may be wrong. As an informal disclaimer, I invoke the wisdom of one adventurous neuroscientist, Warren McCulloch, who said when introducing a promising theory: “Don’t bite my finger, look where I am pointing” (Papert, 1965).

Today, researchers can detail many of the neural mechanisms I mentioned: for instance, how the premotor cortex (PC) prepares the primary motor cortex (PMC) to implement a series of commands for a voluntary movement (e.g., extending a hand) that was prompted by an outside world stimulus (e.g., handling different qualities, weights, textures of hot and cold pressed papers). They can describe how the pre-supplmenatry motor areas (PMAs) prepare the PMC’s implementation plans for grasping a chain of commands, which the brain generates and enacts by means of the PMC (Medina, 2011). Moreover, researchers can trace intentional actions stimulated by “free will neurons” (Kreiman et al., 2014; Talbot, 2014). Supposedly, these neurons trigger the creative freedom I feel in making Neuroart—even as this art renders unpredictable, non-deterministic aspects of free will (Thorp, 1980), willed behavior (Lau et al., 2004), conscious decision-making (Baumeister, 2008; Mele, 2009), and self-initiated actions (Cunnington et al., 2002; Mueller et al., 2007) connecting “creativity across domains” (Kaufman and Baer, 2005).

## Connecting Intentional Acts with Neuroscience of Creativity, Art and Neuroaesthetics

Draw a Venn diagram with three overlapping circles forming a curved triangle in the center. This simple diagram summarizes the relationship between these three sub-specialties in brain science: Neuroscience of Creativity (top circle), Neuroscience of Art (left circle), and Neuroaesthetics (right circle) with Neuroscience of Intentions at the intersection; that’s where the art *of* science (Siler, 1990) overlaps the science *of* art (Ramachandran and Hirstein, 1999; Solso, 2000), in the search to understand creative, intentional actions.

The meanings and intentions or purposes of Neuroart involve connecting and freely interpreting the information gathered from the neuroscience of creativity, art, and neuroaesthetics—stimulating new thoughts on the creative nature of the brain. This artwork ruminates the insightful neuroscientific studies of intention (Lau et al., 2004; Iacoboni et al., 2005; Nakahara and Miyashita, 2005; Cona et al., 2015; Xu et al., 2015) as they relate to the neuroscience of creativity (Jung et al., 2013; Dietrich and Haider, 2014). Collectively, these studies of creative cognition seek to understand what creativity is (Sternberg and Lubart, 1999); how it is manifested in conscious and unconscious intentions (Custers and Aarts, 2010; Simonton, 2010); and how it enhances self-learning, problem-solving and inductive reasoning in work and play (Greenfield et al., 1994). These brain processes are integral to making art (Siler, 1993, 2011; Solso, 1994). After all, art grows out of creative, intentional actions that unite our diverse sense of aesthetics.

One overarching question that’s as relevant to the neuroscience of intentional actions as it is to the neuroscience of creativity, art and neuroaesthetics: Is the creative process the same in art as it is in science or pure and applied mathematics? Consider how the process of creativity uses the same set of cognitive and affective functions to perform various acts of creative seeing and divergent thinking (Siler, 1986; Runco and Richards, 1997; Simonton, 1999), analogical reasoning (Sternberg, 1977; Vartanian, 2012), metaphorical thinking (Lee and Dapretto, 2006), proprioceptive thinking and dimensional thinking (Root-Bernstein, 2011).

Making and appreciating art embody intentional actions, which are not unlike the creative actions in practicing science, as observed in the ArtScience process of discovery and innovation (Root-Bernstein et al., 2011; Siler, 2011). These actions can be connected to elements of creative cognition: “insight,” “convergent” and “divergent” thinking, among other elements commonly associated with intentional actions, creative and critical thinking (Jung et al., 2013), and decision-making involving unconscious, intuitive [fast] and conscious, analytical [slow] thinking (Kahneman, 2011). Case in point: ArtNano innovations addressing global challenges (Siler and Ozin, 2012; Qian et al., 2015).

## Pinpointing Neural Correlates of Creative Cognitions that Manifest Intentional Actions

The brain activities that most interest me as a visual artist concern the use of metaphors and analogies in art and science (Gentner et al., 2001; Chatterjee, 2004, 2011; Shibata et al., 2007; Schmidt and Seger, 2009; Yang et al., 2009). Metaphorical thinking enables us to make intuitive leaps of insight (Siler, 1988, 1997; Holyoak and Thagard, 1995) that move our imagination from an unconscious intention (e.g., wondering whether or not a connection exists between two things) to an intentional action (e.g., forming a hypothesis about the apparent connection and falsifying it using the scientific method). Consider how Albert Einstein leaped metaphorically to “picture what it would be like to ride alongside a light beam,” while composing his Special Theory of Relativity (Einstein, 1905). As the biographer Walter Isaacson (2007) describes: “This type of visualized thought experiment—*Gedankenexperiment*—became the hallmark of Einstein’s career”.

The Nobel laureate chemist and poet Roald Hoffmann has observed: “The images that scientists have as they do science are metaphorical. The imaginative faculty is set in motion by mental metaphor. Metaphor shifts the discourse, not gradually, but with a vengeance. You see what no one had seen before” (Hoffmann, 2006). This versatile connection-making process often inspires the formation of intentions evident in commonplace and exceptional creative cognition (Siler, 1997; Simonton, 1999). Consider the nature-inspired metaphors Leonardo da Vinci wielded like all-purpose tools for discovering nature’s unity; specifically, how “everything connects to everything else” (*A Treatise on Painting* DaVinci, 1452–1519; Rigaud, 2005; Firmin and Siler, 2014).

## Creating, Discovering, Innovating, and Learning Through Neuroart

“*The Brain Theater of Mental Imagery* with *Thought Assemblies* emerging from the Limbic System” (1979–82, 2014; Figure 1) interprets the brain’s creative engine of innovation that connects and integrates the process of consciousness, attention and intention (Lau et al., 2004) with acts of creativity. The art speculates on various neural mechanisms that move the mind from intentional states (e.g., envisioning the design of an immersive, experiential artwork) to voluntary actions and visceral responses (e.g., building the structures I’ve envisioned). While reading about the inferior parietal lobule (IPL) neurons (Fogassi et al., 2005), immediately I imagine how the IPL neurons are activated in this creative process, as I manipulate my pencils in drawing various conceptual and design possibilities for this evolving art installation.

When visitors enter this immersive artwork, they’re enveloped by a womb-like structure that resembles a mid-sagittal section of the limbic system (outlined in red in the top diagram). This structure represents the heart of the brain: a region where thoughts, feelings and emotions meld as they’re integrated and interpreted by the whole brain (Siler, 1986; Feldman et al., 2007) via the web of connections linking the prefrontal cortex and limbic system structures (Boeree, 2011) and providing feedback to the sensory cortices and brain reward areas involving the nucleus accumbens (Salimpoor et al., 2013). It serves as a “Creative Commons,” invoking images of a resource that belongs to and affects the networked community of subcortical systems.

Mounted on the massive cortical screen, “The Brain Theater of Mental Imagery, ” is the multipart “Thought Assemblies.” This artwork consists of 515 constituent images, each one depicting a mental image that’s been rendered on a substrate. The substrate represents the concomitant neural processes corresponding to the creation of the images. Collaged on the surface of “Thought-Assemblies” are examples of historical and everyday innovations that reveal inspired acts of creativity.

**Figure 2:**
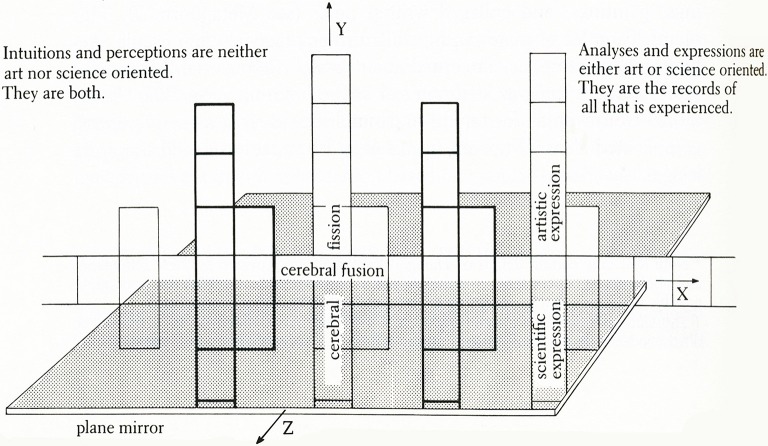
**The Conceptual Armature of Thought-Assemblies—a diagram indicating the information portrayed in this artscience work, which consists of three interactive axes.** Presented on the X-axis is information based on intuition and perception about the brain and universe. Intersecting this plane is the plane mirror, or Z-axis, which reflects vertically above and below the X-axis. Above the X-axis, the information is abstracted and implied, thus entering the realm of art. Below the X-axis, qualifying and quantifying information is added, entering the realm of science. *Thought-Assemblies* indicates that analytic and artistic thought can proceed from the same frame of insight-perception and that these two models of thought converge. As an exercise in topology, if the artwork were folded to form a tube and then the ends of the tube were brought together to form a torus, or donut-shape, the farthest points at both ends of the X- and Y-axes would be continuous.

“Thought Assemblies” envisions and explores the possible structure and organic unity of creative cognition. It examines the neural mechanisms that create and connect thoughts-feelings-actions (Chorover and Chorover, 1982; Shallice, 1988; Kelley et al., 2002), baring the marks of unconscious and conscious intentional actions. In representing brain functioning it combines visual metaphors, physical analogies, symbols, signs, stories, and allegories, which are part of its embodiments, expressions and aesthetics. In fact, it’s a symbolic model of neuropsychological processes that unfolds in Cartesian (X, Y, Z) space (Figure 2). Like any artwork, this one is inescapably self-referential; it presents the personal lexicon, free-associations and perceptions of its creator. As well, it is introspective in that it traces my thought patterns, their contexts, and the perceptual pieces from which these patterns were constructed (Siler, 1986; p. 75).

Metaphorically, this Neuroart interprets *what nature makes and what we make of nature*. It considers how every detail of nature *details* “human nature” (humankind’s integration with nature). The mosaic of drawings and paintings picture the creative process as a system dynamically resembling the fusion and fission processes that form and shape the universe. I hypothesize the mergence of brain functions (“*cerebral fusion*”) at the instant of intuition and the divergence of these functions (“*cerebral fission*”) in moments of analytical reasoning and expression. Here reasoning includes both analytic and affective reasoning in artistic and scientific expressions (representations) of knowledge, experience, etc. Virtually every form or aspect of reasoning is represented in artistic expression including sequential, feature by feature reasoning (as in spatial cognition) and emotional or affective reasoning (i.e., reasoning about and with emotions as in the discriminations of feelings). Figure 2 sums up the overall design of the artwork, which is described in this doctoral dissertation,”Architectonics of Thought: A Symbolic Model of Neuropsychological Processes” (Siler, 1986).

My intention was to present ideas and images towards a theory proper that emerged from a phenomenological study of insight-perception and introspective analyses, relating it to experimental studies of cerebral functions: e.g., evoked potentials (Regan, 1972; Bodis-Wollner, 1982), positron emission tomography (Heiss and Phelps, 1983), and lateralization (Sperry, 1968, 1976; Sperry et al., 1969; Gazzaniga, 1972). I had wanted to verify my hypotheses, but life had other plans for me in the field of Contemporary Art.

I continue to advocate using arts-based learning methods and tools to contribute to neuroscientific discoveries and inspire innovations (Siler, 1997, 2010, 2011; Holman et al., 2007; Root-Bernstein and Root-Bernstein, 1999; Root-Bernstein, 2011; Fetz, 2012; Seifter, 2014). Interpreting big data from the *Brain Activity Map* (BAM) requires broad cross-disciplinary creative collaborations, in order to describe the whole brain’s functional architecture and neural activity. BAM joins the Blue Brain Project (BBP), which maps the labyrinth of synaptic connections between diverse populations of neurons and how they grow. These projects and advancements in Neuroinformatics (Koslow and Subramaniam, 2005) aim to record and decipher “every spike from every neuron” that form the “functional connectome” (Alivisatos et al., 2012), enabling researchers to eavesdrop on the conversations of neurons talking to neurons, and make sense of them in the healthy and diseased brains. Hopefully, this adventurous work will illuminate the unintentional and intentional actions of the creative process in art and science taking us “Closer To Truth” (Kuhn, 2000).

## Summary

The example of Neuroart highlighted here pictures intentional actions. Its contents intimate how the creative process of convergent and divergent thinking is similar in all representations of thought that comprise the interrelated History of Art, Science, Technology, Engineering, Mathematics, and other forms of explicit, implicit and tacit knowledge (Polanyi, 1958/1998, 1967). Neuroart explores ways of experiencing and understanding human creations as metaphorical manifestations of creative and critical thinking that reveal the nature of intentional actions. With that objective, this experimental work aims to catalyze and cultivate innovative thinking in the neuroscience of creativity, which is essential for interpreting anew the neural data being gathered and examined in major relational-data mining endeavors (e.g., http://www.incf.org; BRAIN Initiative,^1^ or Brain Mapping Project; the Human Connectome^2^) (Seung, 2012). These resources already aid researchers in grasping the brain processes of intentions and volitional motor actions that underlie the creative process of making and appreciating art (Kandel, 2012; Ishizu and Zeki, 2011), which include simulating the actions, emotions and sensory impressions we experience as “embodied aesthetics” (Ticini et al., 2015).

## Conflict of Interest Statement

The author declares that the research was conducted in the absence of any commercial or financial relationships that could be construed as a potential conflict of interest.

## Footnotes

^1^http://www.whitehouse.gov/share/brain-initiative

^2^http://www.humanconnectomeproject.org
